# The RNA-binding protein IGF2BP3 is critical for MLL-AF4-mediated leukemogenesis

**DOI:** 10.1038/s41375-021-01346-7

**Published:** 2021-07-29

**Authors:** Tiffany M. Tran, Julia Philipp, Jaspal Singh Bassi, Neha Nibber, Jolene M. Draper, Tasha L. Lin, Jayanth Kumar Palanichamy, Amit Kumar Jaiswal, Oscar Silva, May Paing, Jennifer King, Sol Katzman, Jeremy R. Sanford, Dinesh S. Rao

**Affiliations:** 1 Department of Pathology & Laboratory Medicine, David Geffen School of Medicine, UCLA, Los Angeles, CA 90095 USA; 2 Molecular, Cellular, and Integrative Physiology Interdepartmental Ph.D. Program, UCLA, Los Angeles, CA 90095 USA; 3 Department of Molecular, Cellular and Developmental Biology, UCSC, Santa Cruz, CA 95064 USA; 4 Division of Hematology/Oncology, Department of Medicine, UCLA, Los Angeles, CA 90095 USA; 5 Molecular Biology Interdepartmental Doctoral Program, UCLA, Los Angeles, CA 90095 USA; 6 Department of Biochemistry, All India Institute of Medical Sciences, New Delhi, 110029 India; 7 Department of Pathology, Stanford University School of Medicine, Stanford, CA 94305 USA; 8 Division of Rheumatology, Department of Medicine, UCLA, Los Angeles, CA 90095 USA; 9 UCSC Genomics Institute, Santa Cruz, CA 95064 USA; 10 Jonsson Comprehensive Cancer Center (JCCC), UCLA, Los Angeles, CA 90095 USA; 11 Broad Stem Cell Research Center, UCLA, Los Angeles, CA 90095 USA

**Keywords:** Leukaemia, Cancer stem cells, Oncogenes

## Abstract

Despite recent advances in therapeutic approaches, patients with MLL-rearranged leukemia still have poor outcomes. Here, we find that the RNA-binding protein IGF2BP3, which is overexpressed in MLL-translocated leukemia, strongly amplifies MLL-Af4-mediated leukemogenesis. Deletion of *Igf2bp3* significantly increases the survival of mice with MLL-Af4-driven leukemia and greatly attenuates disease, with a minimal impact on baseline hematopoiesis. At the cellular level, MLL-Af4 leukemia-initiating cells require *Igf2bp3* for their function in leukemogenesis. At the molecular level, IGF2BP3 regulates a complex posttranscriptional operon governing leukemia cell survival and proliferation. IGF2BP3-targeted mRNA transcripts include important MLL-Af4-induced genes, such as those in the *Hoxa* locus, and the Ras signaling pathway. Targeting of transcripts by IGF2BP3 regulates both steady-state mRNA levels and, unexpectedly, pre-mRNA splicing. Together, our findings show that IGF2BP3 represents an attractive therapeutic target in this disease, providing important insights into mechanisms of posttranscriptional regulation in leukemia.

## Introduction

Chromosomal rearrangements of the mixed-lineage leukemia (*MLL*, *KMT2A*) gene are recurrently found in a subset of acute lymphoblastic leukemia (ALL), acute myeloid leukemia (AML), and acute leukemia of ambiguous lineage [1]. Despite recent advances in therapeutic approaches, patients with *MLL*-rearranged leukemia have poor outcomes, high risk of relapse, and show resistance to novel targeted therapies [2, 3]. *MLL* encodes an H3K4 methyltransferase required for hematopoietic stem cell (HSC) development during both embryonic and adult hematopoiesis [4–7]. Many translocation partners for *MLL*, including *AF4 (AFF1)*, encode proteins that regulate transcriptional elongation [8–14]. Of more than 90 translocation fusion partner genes, *MLL-AF4* (*KMT2A-AFF1*) is the most common *MLL* fusion protein in patients [15]. Biologically, *MLL*-*AF4*-driven leukemia is a distinct entity compared to non-MLL-rearranged leukemias, with a unique gene expression profile showing significant overlap with stem cell programs [16–18].

At the posttranscriptional level, emerging evidence suggests a role for microRNAs, RNA-binding proteins (RBP), and other RNA-based mechanisms in regulating gene expression during leukemogenesis [19–21]. We recently identified the oncofetal RBP Insulin like growth factor 2 mRNA binding protein 3 (IGF2BP3) as an important regulator of gene expression in *MLL*-rearranged B-ALL [22]. IGF2BP3 is expressed during embryogenesis, lowly expressed in healthy adult tissues, and strongly reexpressed in cancer cells [23]. Elevated levels of IGF2BP3 expression are correlated with diminished patient survival in many cancers and may be a marker of disease aggressiveness in B-ALL [24–26]. Previously, we determined that overexpression of IGF2BP3 in bone marrow (BM) of mice led to a pathologic expansion of hematopoietic stem and progenitor cells (HSPC). IGF2BP3 interacted with and upregulated oncogenic transcripts (e.g., MYC, CDK6) via the 3′UTR, contributing to the pathologic proliferative phenotype [22]. Together, these studies illuminated a novel role for posttranscriptional gene regulation in the pathologic proliferation of HSPCs.

Experimentally, MLL-AF4-driven leukemogenesis has been studied using a range of in vitro and in vivo models leading to significant progress in our understanding of MLL-rearranged leukemia [16, 27–31]. Here, we explicitly tested the requirement for *Igf2bp3* in a bona-fide model of MLL-Af4-driven leukemogenesis [32]. Deletion of *Igf2bp3* significantly increased survival of MLL-Af4 transplanted mice and decreased the numbers and self-renewal capacity of MLL-Af4 leukemia-initiating cells (LICs). Mechanistically, we found that IGF2BP3 targets and modulates the expression of transcripts encoding regulators of leukemogenesis, through multiple posttranscriptional mechanisms. Together, our findings show that IGF2BP3 is a critical regulator of MLL-AF4-mediated leukemogenesis and a potential therapeutic target in this disease.

## Methods

### Molecular biology assays

ChIP-PCR on RS4;11 and SEM cells were performed as previously described [33]. IGF2BP3 ChIP primer sequences were kindly provided by Dr. James Mulloy (University of Cincinnati) [32]. Protein and mRNA extracts were prepared, and western blot/RT-qPCR performed as previously described [34]. Primers and antibodies are listed in Table S1.

### Plasmids, retroviral transduction and BM transplantation (BMT)

The MSCV-MLL-FLAG-*Af4* plasmid was kindly provided by Michael Thirman (University of Chicago) through MTA [32]. Nontargeting (NT) or *Igf2bp3* sgRNA was cloned into an in-house MSCV-hU6-sgRNA-EFS-mCherry vector [35]. Retroviral transduction and BMT are previously described [34, 36]. 5-FU enriched BM and Lin− cells were spin-infected four times with MSCV-*MLL*-FLAG-*Af4* virus at 30 °C for 45 min with polybrene and selected with 400 μg/ml G418 for 7 days. MLL-Af4 Cas9-GFP cells were retrovirally infected with MSCV-hU6-sgRNA-EFS-mCherry.

### Mice

C57BL/6J and B6J.129(Cg)-Gt(ROSA)26Sor^tm1.1(CAG-cas9*,-EGFP)Fezh^/J (Cas9-GFP BL/6J) mice were from Jackson Laboratory. The UCI Transgenic Mouse Facility utilized CRISPR-Cas9 to insert loxP sites flanking exon 2 of *Igf2bp3* to generate *Igf2bp3*^f/f^ mice. To generate conditional KO, *Igf2bp3*^f/f^ mice were bred with Vav1-Cre mice. Consistent with prior reports, this strategy led to “leaky” Cre expression, resulting in germline deletion [37–39]. To isolate floxed and deletion (del) alleles, mice were back-crossed onto C57BL/6 mice with successful germline, Mendelian transmission of del and floxed alleles in two successive generations (Table S2). Mice heterozygous for del allele were mated, leading to homozygous *Igf2bp3* deletion and *Igf2bp3*^del/del^ mice (I3KO) used in this study. Blinding or randomization was not applied to mice experiments.

### Cell culture and flow cytometry

RS4;11, SEM, 70Z/3, and HEK293T cell lines were cultured as previously described [34]. Lin− cells were cultured in IMDM with 15% FBS supplemented with SCF, IL-6, FLT3, and TPO. CD11b+ cells were isolated from splenic tumors for positive selection by MACS (Miltenyi). Blood, BM, thymus, and spleen were collected from mice at indicated time points and staining performed as previously described [22]. Antibodies are provided in Table S1. Flow cytometry was performed on a BD FACS LSRII and analysis using FlowJo software.

### Histopathology

Fixation, sectioning, and analysis were performed as previously described (DSR) [36].

### Competitive repopulation assay and secondary leukemia transplantation

Competitive repopulation experiments are previously described [22]. For leukemia transplantation, BM was collected from WT/MLL-Af4 or I3KO/MLL-Af4 mice that succumbed to leukemia at 10–14 weeks post transplantation and injected into 8-week-old immunocompetent CD45.1+ female mice.

### RNA-seq

Single-end, strand-specific RNA sequencing was performed on Illumina HiSeq3000 for Lin− and CD11b+ samples, 15–20 million reads/sample (UCLA Technology Center for Genomics & Bioinformatics). Analysis is previously described [22]. RNA-seq reads were mapped to the mouse genome assembly mm10 using STAR version X. Repeat sequences were masked using Bowtie 2 [40] and RepeatMasker [41]. Differentially expressed genes (DEGs) were identified using DESeq2 [42] (CD11b+) and fdrtool [43] (Lin−). Multiple testing correction used the Benjamini–Hochberg method. Significant DEGs have adjusted *P* value < 0.1 and log2FC > 1. Data collection and parsing were completed with bash and python2.7. Statistical analyses were performed using R version 3.5.1. Enrichment analyses were completed with Metascape [44] and gene set enrichment analysis (GSEA) using GSEAPreranked after π-value calculation [45–47].

### Alternative splicing estimation

Mixture of Isoforms (MISO) Bayesian inference model v0.5.4 with mm10 “exon-centric annotation” quantified alternative splicing events [48]. Percent spliced in (PSI) was quantified for each event by number of read counts supporting both events and unique reads to each isoform. Delta PSI was calculated by subtracting from WT. Significant differential events had delta PSI > 0.1, Bayes factor ≥ 10, and sum of exclusion and inclusion reads ≥ 10.

### Enhanced crosslinking-immunoprecipitation (eCLIP)

eCLIP was completed on a minimum of two biological replicates with two technical replicates and size matched input (smInput) samples (Eclipse BioInnovations). Overall, 5 × 10^5^ cells were UV crosslinked (245 nm, 400 mJoules/cm^2^), RNAse I treated, and immunoprecipitated with anti-IGF2BP3 antibody (MBL RN009P) coupled to magnetic Protein G beads. Paired-end RNA-seq was performed on Illumina HiSeq4000 (UCSF Genomics Core Facility). Peaks were called using CLIPper [49] and filtered on smInput (FS1). HOMER [50] annotatePeaks.pl and findMotifs.pl provided peak genomic locations and motif enrichment. Background for peaks within DEGs was simulated using bedtools [51] and shuffled 1000 times.

### Statistics

Data represent mean ± SD for continuous numerical data, unless otherwise noted in figure legends. One-way ANOVA followed by Bonferroni’s multiple comparisons test (>2 groups) or two-tailed Student’s *t* tests were performed using GraphPad Prism software.

## Results

### IGF2BP3 is integrated into the MLL-AF4 transcriptional program

**Fig. 1:**
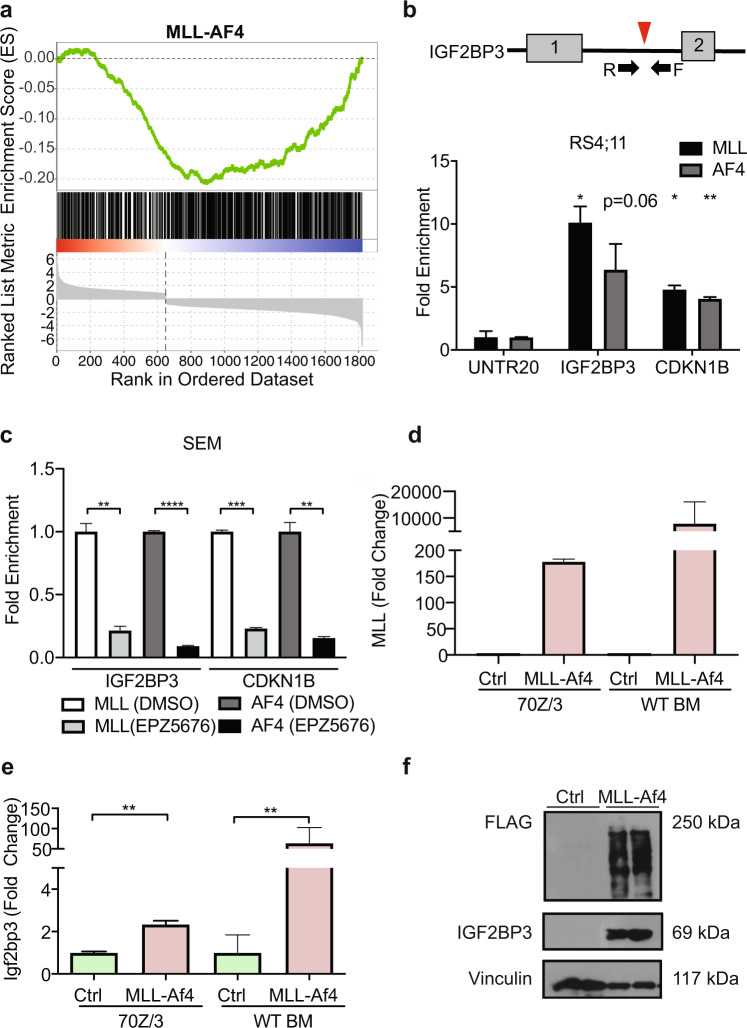
MLL-AF4 transcriptionally induces IGF2BP3. **a** GSEA of differentially expressed genes from IGF2BP3 depleted RS4;11 cells shows significant negative enrichment with MLL-AF4 ChIP targets (nominal *P* value: 0.001, FDR: 0.001, Normalized ES: −1.54)). **b** Schematic of MLL-AF4 binding site in intron 1 of IGF2BP3 (top). ChIP-qPCR shows fold enrichment for IGF2BP3 and CDKN1B with MLL and AF4 IP in RS4;11. Normalized to UNTR20, an untranscribed region (*t* test; *P < 0.05, **P < 0.01). **c** Fold enrichment from ChIP-qPCR of SEM cells show reduced binding of MLL-AF4 to IGF2BP3 with treatment of the DOT1L inhibitor, EPZ5676. Normalized to DMSO. (*t* test; **P < 0.01, ***P < 0.001, ****P < 0.0001). **d** Expression of MLL through RT-qPCR of 70Z/3 transduced with either control (Ctrl) or MLL-Af4 vector selected with G418 and MLL expression at the RNA level in the BM of WT recipients transplanted with Ctrl or MLL-Af4 HSPCs. **e** Induction of *Igf2bp3* at the RNA level in selected 70Z/3 with MLL-Af4 and in the BM of WT recipients transplanted with Ctrl or MLL-Af4 HSPCs (bottom) (*t* test; **P < 0.01). **f** Induction of IGF2BP3 at the protein level in BM from mice transplanted with MLL-Af4-transduced WT donor HSPCs.

To understand the overlap of transcriptional and posttranscriptional regulation in *MLL*-rearranged leukemia, we compared IGF2BP3-regulated targets with a published MLL-Af4 ChIP-Seq dataset [22, 32]. Transcripts modulated by IGF2BP3 were significantly enriched for MLL-Af4-bound genes (Fig. 1a; Supplementary Fig. 1a). Interestingly, IGF2BP3 itself was a direct transcriptional target of MLL-Af4, with binding sites within the first intron and promoter region (Supplementary Fig. 1b) [32]. To confirm, we performed ChIP-PCR assays on RS4;11 and SEM, human MLL-AF4 translocated B-ALL cell lines, and determined that the first intron of *IGF2BP3* is strongly bound by MLL-AF4 (Fig. 1b; Supplementary Fig. 1c). This MLL-AF4 binding was abrogated when SEM cells were treated with the DOT1L inhibitor, EPZ5676, and the bromodomain inhibitor, IBET-151 (Fig. 1c; Supplementary Fig. 1d) [52]. Furthermore, we observed an MLL-AF4-dose-dependent increase in luciferase reporter activity, using the promoter region upstream of the IGF2BP3 transcription start site (Supplementary Fig. 1e). In the murine pre-B 70Z/3 cell line and primary murine BM cells, transduction with retroviral MLL-Af4) [32] caused an ~64-fold upregulation of *Igf2bp3* mRNA (Fig. 1d, e). Concordantly, IGF2BP3 protein was upregulated in MLL-Af4-transduced primary BM cells (Fig. 1f). Furthermore, enforced expression of another MLL fusion protein, MLL-AF9, and other non-MLL leukemia drivers, including AML1-ETO, MYC, and NRAS in primary HSPCs, show that the upregulation of *Igf2bp3* is specific to MLL-Af4 (Supplementary Fig. 1f). These findings of *Igf2bp3* specificity are in line with those that we and others have previously reported, as well as in publicly available datasets [22, 26, 53]. Interestingly, induced expression of structurally and functionally related paralogs *Igf2bp1* and *Igf2bp2* was noted with enforced expression of non-MLL-Af4 oncogenic drivers, again in concordance with observations in human leukemia (Supplementary Fig. 1g, h) [22, 26, 54]. Taken together, these findings demonstrate that MLL-Af4 specifically drives the expression of *Igf2bp3* in vivo.

### Normal hematopoiesis is maintained in Igf2bp3 KO mice

To test the in vivo requirement for IGF2BP3 in leukemogenesis, we generated an *Igf2bp3* KO (I3KO) mouse. We initially generated a floxed *Igf2bp3* allele (f/f; Supplementary Fig. 2a) using CRISPR-Cas9. In the course of mating these mice with Vav1-Cre mice, we serendipitously generated a germline knockout allele (del), which we isolated and characterized (Supplementary Fig. 2b). This has been previously reported in the Vav1-Cre mouse strain, which displays “leaky” Cre expression resulting in germline deletion [37–39]. Mendelian inheritance was confirmed for the isolated germline del allele, although distribution of genotypes was marginally skewed (Table S2). Deletion of *Igf2bp3* was confirmed at the DNA, RNA, and protein level (Supplementary Fig. 2c–e). These *Igf2bp3*^*del/del*^ (I3KO) mice were used in this study. Immunophenotyping of I3KO mice showed no significant differences in numbers of HSPCs in the BM compared to WT (Supplementary Fig. 2f). I3KO mice showed similar numbers of myeloid-lineage progenitors (CMPs, GMPs, and MEPs) (Supplementary Fig. 2g), normal B-cell development [55] (Supplementary Fig. 2h), and normal numbers of mature B lymphoid, T lymphoid, and myeloid lineages in the BM and spleen (Supplementary Fig. 2i, j). Hence, I3KO mice demonstrate preserved normal, steady-state adult hematopoiesis.

### Igf2bp3 deletion increases the latency of MLL-Af4 leukemia and survival of mice

**Fig. 2:**
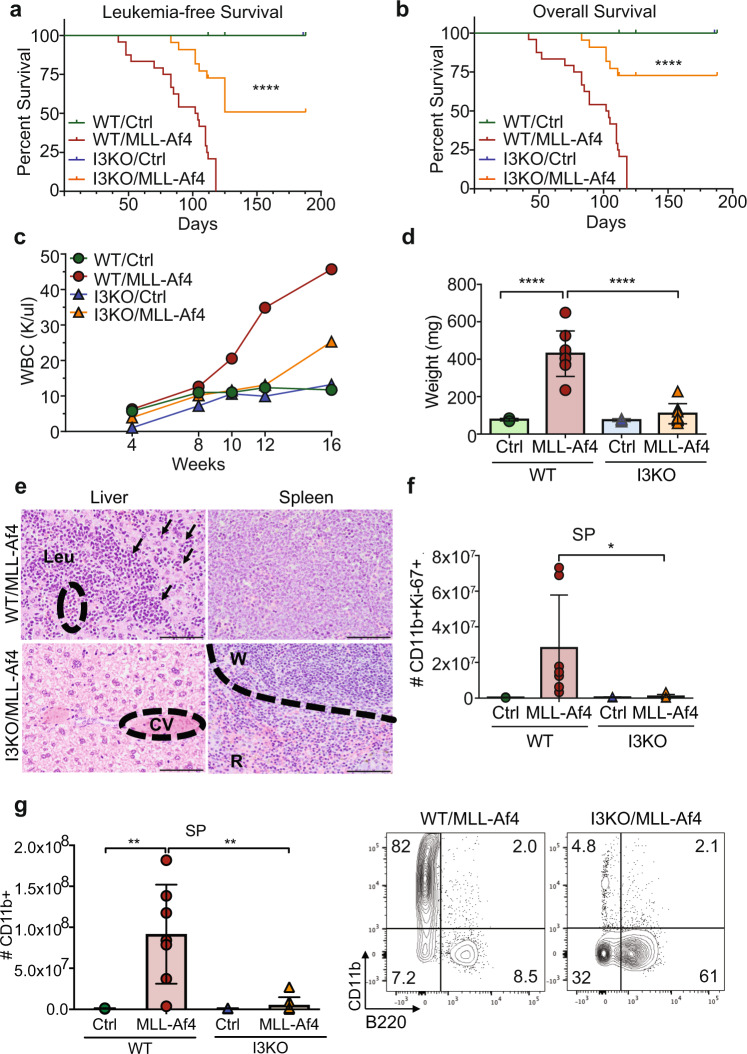
Igf2bp3 deletion delays leukemogenesis and reduces disease severity. **a** Leukemia-free survival of mice transplanted with control (Ctrl) or MLL-Af4-transduced HSPCs from WT or Igf2bp3 KO mice (Kaplan–Meier method with log-rank test; ****P < 0.0001). **b** Overall survival of mice transplanted with Ctrl or MLL-Af4-transduced HSPCs from WT or I3KO mice (*n* = 12 WT/Ctrl, *n* = 24 WT/MLL-Af4, *n* = 7 I3KO/Ctrl, *n* = 22 I3KO/MLL-Af4; Kaplan–Meier method with log-rank test; ****P < 0.0001). **c** Time course of WBC in the PB of mice transplanted with Ctrl or MLL-Af4-transduced HSPCs from WT or I3KO mice (data represented as means of three experiments; *n* = 4 Ctrl, *n* = 8 MLL-Af4 per experiment). **d** Spleen weights of mice transplanted with Ctrl or MLL-Af4-transduced HSPCs from WT or I3KO mice at 14 weeks (*n* = 4 Ctrl, *n* = 8 MLL-Af4; one-way ANOVA followed by Bonferroni’s multiple comparisons test; ****P < 0.0001). **e** H&E staining of liver and spleen of mice transplanted with mice transplanted with MLL-Af4-transduced HSPCs from WT or I3KO mice at 14 weeks. Scale bar: 100 μm; CV central vein; W white pulp; R red pulp; Leu leukemia; arrows showing infiltration. **f** Quantitation of CD11b+Ki67+ cells in the spleen at 14 weeks post transplantation (*n* = 4 Ctrl, *n* = 8 MLL-Af4; one-way ANOVA followed by Bonferroni’s multiple comparisons test; *P < 0.05). **g** (Left) Number of CD11b+ in the SP of recipient mice that received Ctrl or MLL-Af4-transduced HSPCs from WT or I3KO mice at 14 weeks (one-way ANOVA followed by Bonferroni’s multiple comparisons test; **P < 0.01). (Right) Corresponding representative FACS plots showing CD11b+ and B220+ cells in the SP.

Next, we queried MLL-Af4-mediated leukemogenesis in I3KO mice, utilizing BMT (Supplementary Fig. 3a). Retroviral MLL-Af4 transduction was equivalent between WT and I3KO donor BM, based on DNA copy number (Supplementary Fig. 3b) and western blot analysis (Supplementary Fig. 3c). Following transplantation of transduced HSPCs, *Igf2bp3* loss significantly increased both leukemia-free and overall survival of MLL-Af4 mice (Fig. 2a, b). The median survival of I3KO/MLL-Af4 mice was greater than 157 days, compared to 103 days for control mice. White blood cell (WBC) and myeloid cell counts in I3KO/MLL-Af4 mice were significantly reduced, compared with the control mice (Fig. 2c; Supplementary Fig. 3d). On average, I3KO/MLL-Af4 mice became overtly leukemic much later than the control mice peripheral blood (112 versus 70 days) (Fig. 2c). Concordantly, peripheral blood smears showed reduced circulating blasts in I3KO/MLL-Af4 mice (Supplementary Fig. 3e). Together, these findings indicated that *Igf2bp3* is required for efficient MLL-Af4-mediated leukemogenesis.

### Igf2bp3 modulates disease severity in MLL-Af4-driven leukemia

The MLL-Af4 model utilized here causes a highly penetrant, aggressive form of leukemia in mice. In timed experiments, I3KO/MLL-Af4 transplanted mice showed a highly significant, approximately fourfold reduction in spleen weights at 14 weeks post transplant compared to WT/MLL-Af4 mice (Fig. 2d). I3KO/MLL-Af4 mice showed reduced infiltration of the spleen and liver by leukemic cells, which obliterated normal tissue architecture in WT/MLL-Af4 mice (Fig. 2e). In line with this, I3KO/MLL-Af4 transplanted mice showed a significant reduction in CD11b+ cells, which were less proliferative (CD11b+Ki67+), both in the spleen (~30-fold) and BM (~2.5-fold) at 14 weeks (Fig. 2f, g; Supplementary Fig. 3f, g). Thus, *Igf2bp3* deletion significantly reduces tumor burden and attenuates disease severity in MLL-Af4 transplanted mice.

### Igf2bp3 is required for LIC function in vitro

**Fig. 3:**
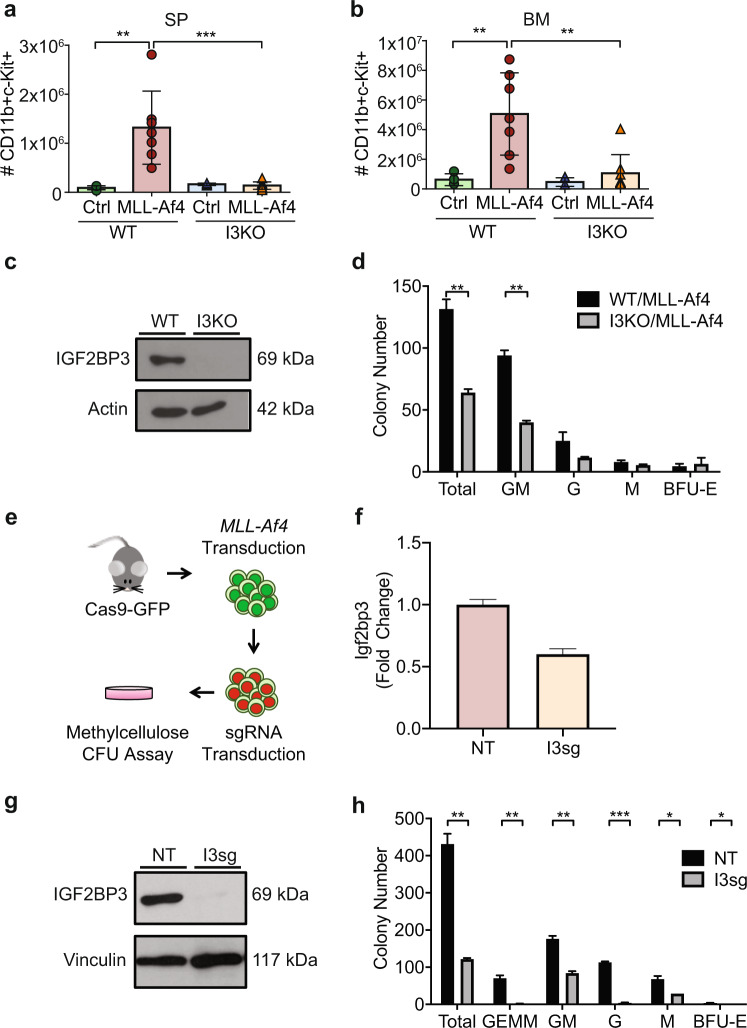
*Igf2bp3* is required for LIC function in endpoint colony formation assays. **a** Quantification of CD11b+c-Kit+ cells in the spleen of recipient mice at 14 weeks post transplantation (*n* = 4 Ctrl, *n* = 8 MLL-Af4; one-way ANOVA followed by Bonferroni’s multiple comparisons test; **P < 0.01). **b** Quantitation of CD11b+c-Kit+ cells in the BM 14 weeks post transplantation (*n* = 4 Ctrl, *n* = 8 MLL-Af4; one-way ANOVA followed by Bonferroni’s multiple comparisons test; **P < 0.01, ***P < 0.001). **c** Expression of IGF2BP3 of in WT/MLL-Af4 and I3KO/MLL-Af4 immortalized Lin− cells at the protein level. **d** Colony formation assay of WT/MLL-Af4 and I3KO/MLL-Af4 immortalized Lin− cells (*t* test; **P < 0.01). **e** Schematic of collection of Cas9-GFP MLL-Af4 Lin− cells and CRISPR-Cas9-mediated deletion of *Igf2bp3*. **f** Expression of Igf2bp3 in Cas9-GFP MLL-Af4 Lin− cells in nontargeting (NT) and Igf2bp3 deleted (I3sg) cells by RT-qPCR. **g** Expression of IGF2BP3 in NT and I3sg Cas9-GFP MLL-Af4 Lin− cells at the protein level. **h** Colony formation assay of NT and I3sg deleted Cas9-GFP MLL-Af4 Lin− cells (*t* test; *P < 0.05, **P < 0.01, ***P < 0.001).

Several studies highlight the importance of LICs in both human and mouse leukemia. In the MLL-Af4 model, LICs show expression of CD11b and c-Kit [17, 32, 56]. Given our findings of delayed initiation and decreased disease severity, we characterized these LICs (CD11b+c-Kit+) in I3KO/MLL-Af4 transplanted mice, finding a significant tenfold decrease in numbers in the spleen and fivefold decrease in the BM at 14 weeks (Fig. 3a, b). After confirming deletion of IGF2BP3 at the protein level in immortalized HSPCs (Lin−) from WT/MLL-Af4 and I3KO/MLL-Af4 mice, we turned to endpoint colony-forming unit assays (CFU) to characterize MLL-Af4 LIC dependence on IGF2BP3 (Fig. 3c). Deletion of *Igf2bp3* resulted in an approximately twofold reduction in total colonies and a significant decrease in CFU-GM progenitors (Fig. 3d). To confirm this, we utilized an orthogonal method for CRISPR-Cas9-mediated *Igf2bp3* deletion. Briefly, Lin− cells from Cas9-GFP mice were transduced with MSCV-MLL-Af4 virus. After selection, MLL-Af4 Cas9-GFP Lin− cells were transduced with a retroviral vector containing either a NT sgRNA or sgRNA targeting *Igf2bp3* (I3sg) (Fig. 3e). Importantly, *Igf2bp3* is deleted after MLL-Af4 transformation, a distinction from the prior method (Fig. 3f, g). Deletion of *Igf2bp3* led to a significant reduction in total colony numbers and various colony morphologies (Fig. 3h). The differences in overall colony-forming capacity between the two systems are likely the result of utilizing different methodologies, but in both systems, *Igf2bp3* deficiency led to decreased colony formation.

### *Igf2bp3* is necessary for the function of *MLL-Af4* LICs in vivo

**Fig. 4:**
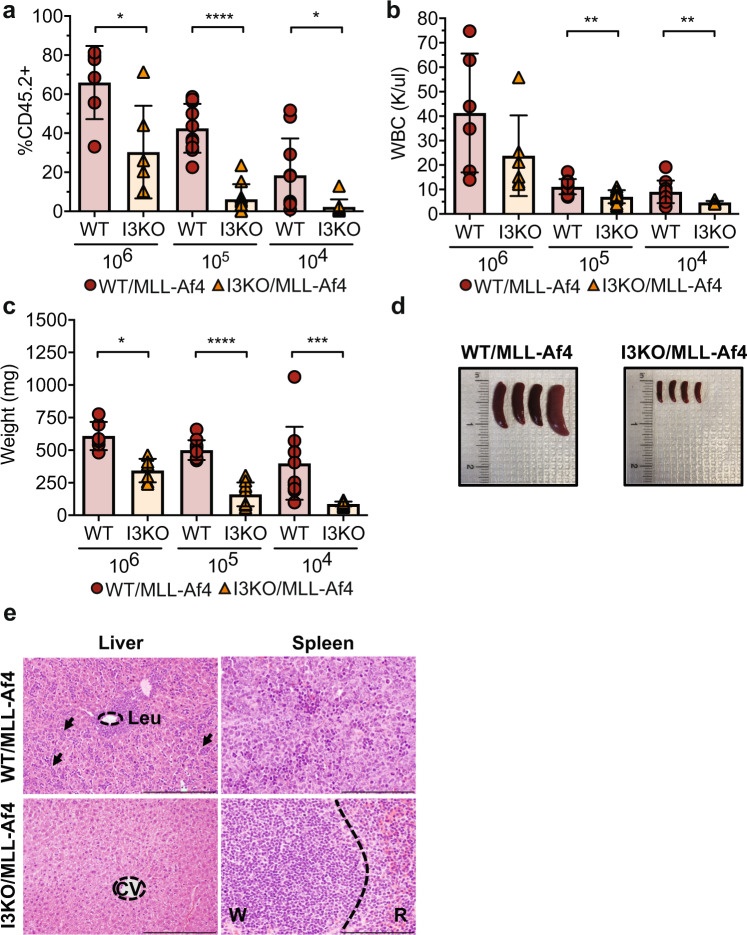
*Igf2bp3* deletion is necessary for MLL-Af4 leukemia-initiating cells to reconstitute mice in vivo. **a** Percentage of CD45.2+ in the peripheral blood of secondary transplanted mice from leukemic WT/MLL-Af4 or I3KO/MLL-Af4 donor mice at 10^6^, 10^5^, and 10^4^ BM cells at 4 weeks post transplantation (For all panels in this figure: *n* = 6 recipient mice per genotype for 10^6^ cells and  *n* = 10  recipient mice per genotype for 10^5^ and 10^4^ cells; *t* test; *P < 0.05, ***P < 0.001, ****P < 0.0001). **b** WBC from PB of secondary transplanted mice from WT/MLL-Af4 or I3KO/MLL-Af4 BM 3–4 weeks post transplant (*t* test; **P < 0.01). **c** Splenic weights of secondary transplanted mice at 4–5 weeks (*t* test; *P < 0.05, ***P < 0.001, ****P < 0.0001). **d** Images of splenic tumors in secondary mice transplanted with 10,000 BM cells from WT/MLL-Af4 mice (left) or I3KO/MLL-Af4 mice (right) at 5 weeks. **e** H&E staining of liver and spleen of secondary transplant recipients that received 10^5^ cells at 4 weeks. Scale bar: liver, 200 μm; spleen, 100 μm; CV central vein, W white pulp, R red pulp, Leu leukemia; arrows showing infiltration.

Since *Igf2bp3* deletion reduces LIC numbers and impairs LIC function, we next determined if *Igf2bp3* affects LIC capability to initiate MLL-Af4 leukemia in vivo. First, to investigate baseline HSC function in I3KO mice, we completed competitive repopulation BMT by transplanting lethally irradiated CD45.1 recipient mice with 50% of WT or I3KO CD45.2 donor BM and 50% CD45.1 donor BM. We found no defect in engraftment over time in I3KO recipients (Supplementary Fig. 4a). Moreover, we determined no differences in multilineage hematopoietic reconstitution ability of I3KO donor cells, as immature lineages in the BM and mature lineages in the periphery were intact (Supplementary Fig. 4b–h). With no baseline differences in reconstitution by normal HSPCs, we investigated if *Igf2bp3* impacted the number of effective LICs in secondary transplantation. Equal numbers (10^6^, 10^5^, and 10^4^) of leukemic BM cells from WT and I3KO mice were transplanted into immunocompetent CD45.1 mice. At 4 weeks post transplantation, mice that received 10^6^ I3KO/MLL-Af4 cells had significantly reduced donor CD45.2+ engraftment (Fig. 4a). With 10^5^ and 10^4^ cells, we no longer observed measurable leukemic burden in recipient mice (Fig. 4a), suggesting that LIC active cell frequency in I3KO/MLL-Af4 mice is lost between 10^6^ and 10^5^ cells (Fig. 4a) [57]. WBC and splenic weights were significantly decreased in I3KO/MLL-Af4 transplanted mice (Fig. 4b–d). Histologically, leukemic infiltration was absent in the spleen and liver of 10^5^ I3KO/MLL-Af4 transplanted mice (Fig. 4e). Thus, *Igf2bp3* deletion results in significant reduction in reconstitution of MLL-Af4 transplanted mice, suggesting that *Igf2bp3* is necessary for the self-renewal capability of LICs in vivo.

### IGF2BP3 supports oncogenic gene expression networks in LIC-enriched and bulk leukemia cells

**Fig. 5:**
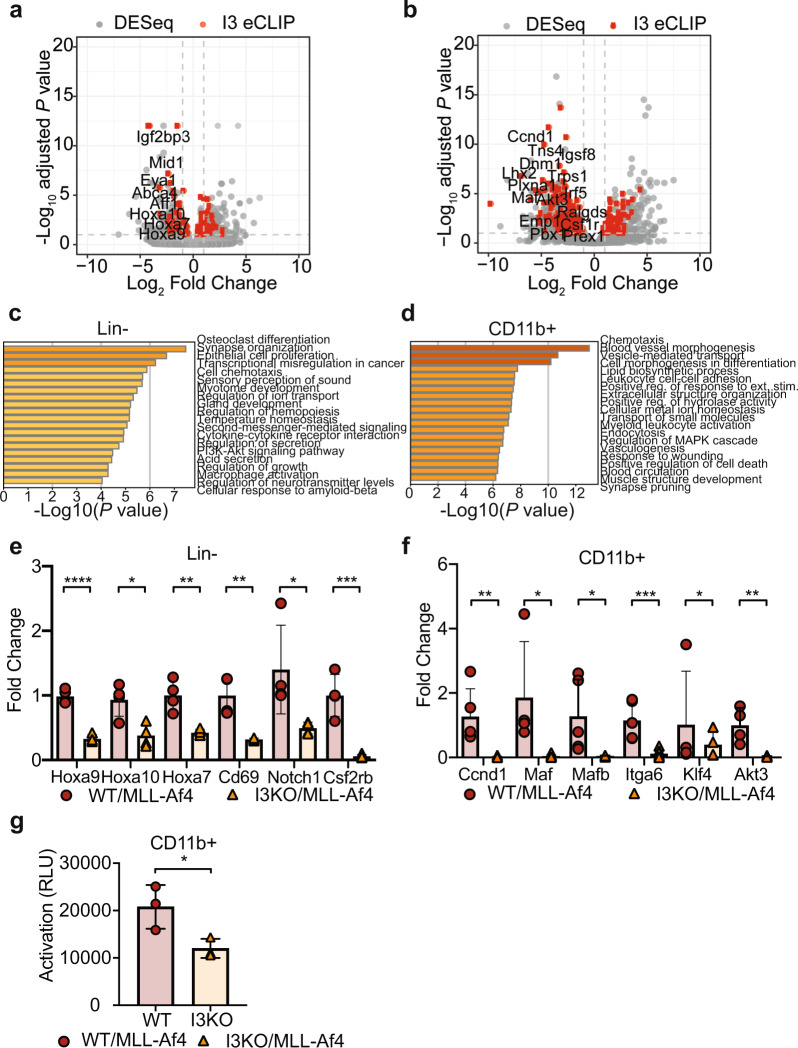
IGF2BP3 enhances MLL-Af4-mediated leukemogenesis through targeting transcripts within leukemogenic and Ras signaling pathways. **a** Volcano plot of differentially expressed genes determined using DESeq analysis on RNA-seq samples from WT/MLL-Af4 or I3KO/MLL-Af4 Lin− cells. Dotted lines represent onefold change in expression (vertical lines) and adjusted *P* < 0.1 cutoff (horizontal line). IGF2BP3 eCLIP-seq targets are highlighted in red. **b** Volcano plot of differentially expressed transcripts determined using DESeq analysis on RNA-seq samples from WT/MLL-Af4 or I3KO/MLL-Af4 CD11b+ cells. Dotted lines represent onefold change in expression (vertical lines) and adjusted *P* < 0.1 cutoff (horizontal line). IGF2BP3 eCLIP-seq targets are highlighted in red. **c** GO Biological Processes and KEGG pathway enrichment determined utilizing the Metascape enrichment analysis webtool on MLL-Af4 Lin− IGF2BP3 DESeq dataset with an adjusted *P* < 0.05 cutoff. **d** GO biological processes and KEGG pathway enrichment determined utilizing the Metascape enrichment analysis webtool on MLL-Af4 CD11b+ IGF2BP3 DESeq dataset with an adjusted *P* < 0.05 cutoff. Bar graphs are ranked by *P* value and overlap of terms within gene list. **e** Expression of leukemogenic target genes in WT/MLL-Af4 and I3KO/MLL-Af4 Lin− cells by RT-qPCR (*n* = 4; *t* test; *P < 0.05, **P < 0.01, ****P < 0.0001). **f** Expression of Ras signaling pathway genes in WT/MLL-Af4 and I3KO/MLL-Af4 CD11b+ cells by RT-qPCR (*n* = 4; *t* test; *P < 0.05, **P < 0.01, ***P < 0.001). **g** Ras GTPase activity by ELISA in WT/MLL-Af4 and I3KO/MLL-Af4 CD11b+ cells (*n* = 3; *t* test; *P < 0.05).

To identify differentially expressed transcripts, we sequenced RNA from WT/MLL-Af4 and I3KO/MLL-Af4 Lin− and CD11b+ bulk leukemia cells after confirming expression of MLL and *Igf2bp3* (Fig. 3c; Supplementary Fig. 5a–e). Differential expression analysis by DEseq2 revealed 208 upregulated and 418 downregulated transcripts in CD11b+ cells, and 189 upregulated and 172 downregulated transcripts in Lin− cells (Fig. 5a, b; Tables S3 and 4) [42]. We identified a significant enrichment in transcripts associated with the KEGG term transcriptional misregulation in cancer in both datasets, using Metascape for enrichment analyses [44] (Fig. 5c, d). Interestingly, discrete oncogenic signaling pathways were enriched in Lin− (PI3K/AKT) and CD11b+ cells (GTPase, MAPK pathway) (Fig. 5c, d). This was confirmed by GSEA, with significant enrichment for the Hallmark KRAS pathway in CD11b+ cells (Supplementary Fig. 5f) and GO Oxidative phosphorylation in Lin− cells (Supplementary Fig. 5g). To validate the RNA-seq data in Lin− cells, we focused on enriched differentially regulated genes including *Csf2rb, Notch1*, *Cd69*, and *Hoxa* cluster of transcripts, including *Hoxa9*, *Hoxa10*, and *Hoxa7*. We observed a significant decrease in steady-state mRNA levels for these transcripts in I3KO/MLL-Af4 Lin− cells by RT-qPCR (Fig. 5e). In I3KO/MLL-Af4 CD11b+ cells, we confirmed that transcripts encoding *Ccnd1*, *Maf*, *Mafb, Itga6, Klf4*, and *Akt3* were decreased (Fig. 5f). Furthermore, we determined that there was a significant decrease in Ras GTPase activity in I3KO cells by ELISA assay (Fig. 5g). Together, these data demonstrate that IGF2BP3 plays a major role in amplifying the expression of many cancer-related genes in Lin− and CD11b+ cells.

### eCLIP analysis reveals a putative role for IGF2BP3 in precursor mRNA (pre-mRNA) splicing

**Fig. 6:**
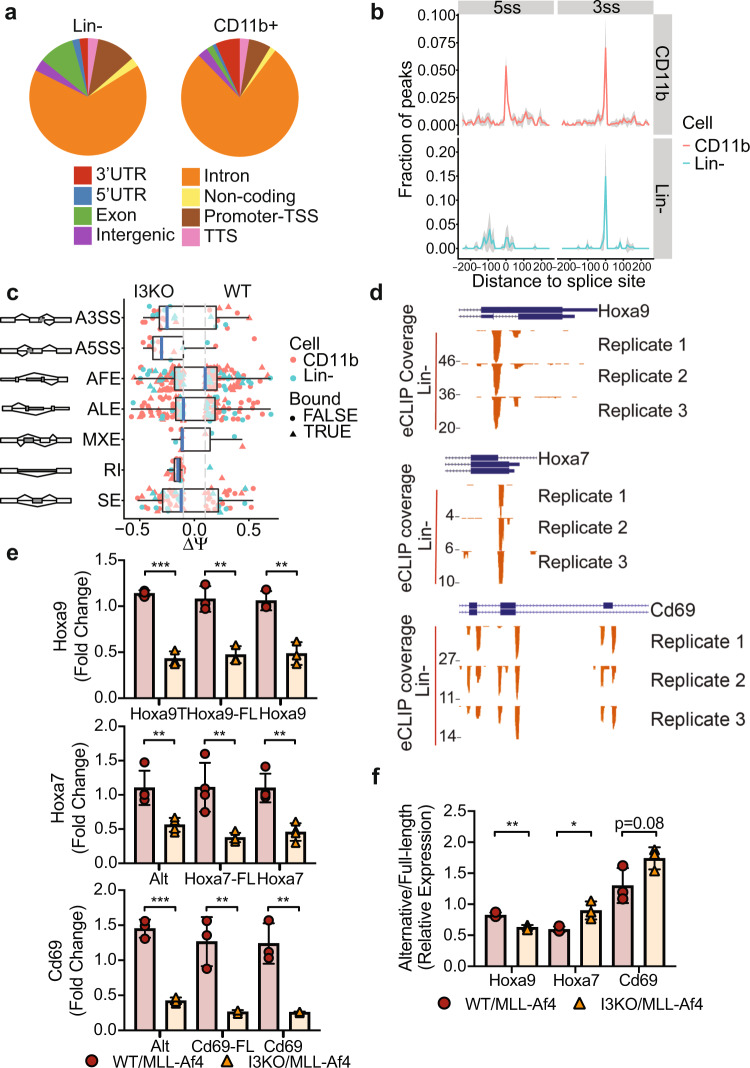
eCLIP analysis reveals IGF2BP3 function in regulating alternative pre-mRNA splicing. **a** Genomic locations of IGF2BP3 eCLIP peaks in WT/MLL-Af4 Lin− cells and CD11b+ cells. Cell type differences in location of exonic peaks were noted: in CD11b+ cells, a greater proportion of exonic peaks were found in 3′UTRs, whereas a greater proportion of peaks mapped to internal exons in Lin− cells. **b** Histogram showing normalized IGF2BP3 eCLIP peak counts and distance from IGF2BP3 eCLIP peak of 5′ (5ss) and 3′ (3ss) splice sites in WT/MLL-Af4 CD11b+ (top) cells and Lin− cells (bottom). **c** Distribution of types of alternative splicing patterns for WT/MLL-Af4 or I3KO/MLL-Af4 Lin− and CD11b+ cells using MISO analysis. Delta psi values plotted indicate difference in isoforms. The event types included are the following, with abbreviations: A3SS alternative 3′ splice sites, A5SS alternative 5’ splice sites, AFE alternative first exons, ALE alternative last exons, MXE mutually exclusive exons, RI retained introns, SE skipped exons, Bound IGF2BP3 eCLIP target. **d** UCSC Genome Browser snapshots of the Hoxa9, Hoxa7, and Cd69 loci. Each panel shows the exon–intron structure of the gene and unique read coverage from three eCLIP biological replicates from WT/MLL-Af4 Lin− cells. The maximum number of reads at each position is indicated to the left of each histogram. **e** Expression of Hoxa9, Hoxa7, and Cd69 splice variants in WT/MLL-Af4 and I3KO/MLL-Af4 Lin− cells by RT-qPCR utilizing primers which detect only its respective alternative splice isoforms (Hoxa9T, Alt), full-length isoforms (-FL), and both isoforms (*n* = 3–4; *t* test; **P < 0.01, ***P < 0.001). **f** Relative expression ratio of alternative splice isoform to full-length isoform (alternative/full-length) in WT/MLL-Af4 and I3KO/MLL-Af4 Lin− cells by RT-qPCR (*n* = 3; *t* test; *P < 0.05, **P < 0.01).

To determine how IGF2BP3 modulates gene expression in MLL-Af4 leukemia, we performed eCLIP-seq (Fig. 5a, b; Tables S3 and 4; Supplementary Fig. 6a). We found that a significant fraction of the differentially expressed mRNAs are bound by IGF2BP3 (Supplementary Fig. 6b). Motif analysis confirmed an enrichment of CA-rich elements (Supplementary Fig. 6c) [58]. Although the majority of peaks were present within introns, we observed cell type-specific differences in the locations of exonic IGF2BP3 binding sites (Fig. 6a). The eCLIP data revealed numerous peaks within pre-mRNA in both Lin− and CD11b+ cells, suggesting a potential role in splicing regulation. To characterize this observation, we utilized MISO analysis to identify differentially spliced transcripts [48]. Across both cell lines, we identified hundreds of transcripts with IGF2BP3-dependent changes in alternative splicing, including 97 differential splicing events in Lin− and 261 splicing events in CD11b+ cells (Supplementary Fig. 6d). After merging all replicate eCLIP data for each cell type, we determined the position of eCLIP peaks relative to splice sites for splicing events identified by MISO (Fig. 6b). Most event types exhibited both increases and decreases in PSI, whereas intron retention (RI) events showed a consistent reduction in splicing in the I3KO/MLL-Af4 cells (Fig. 6c). A significant fraction of alternatively spliced transcripts contained IGF2BP3 binding sites in proximity of the splicing event (Supplementary Fig. 6e), strongest near the 3′ splice site (3′ss), with additional signal near the 5′ splice site. This pattern was observed for each distinct splicing event class that MISO identified, with retained introns exhibiting the strongest bias towards the 3′ss (Supplementary Fig. 6f). Notably, this positional bias in the data was noted for differentially expressed MLL-Af4 target genes, such as *Hoxa9*, *Hoxa7*, and *Cd69* (Fig. 6d). To understand the impact on isoform-specific expression, RT-qPCR primers were designed to nonspecifically detect multiple isoforms or to specifically detect alternatively spliced isoforms (shorter isoforms) and full-length isoforms. As an example, reductions in both isoforms (full-length *Hoxa9* and truncated *Hoxa9T* [59]), as well as in the total level of Hoxa9, were observed in I3KO cells (Fig. 6e). Similar reductions were observed in all isoforms for *Hoxa7* and *Cd69* (Fig. 6e). Furthermore, there was an alteration in the ratio of the alternative to full-length isoform for all three genes (Fig. 6f), highlighting an effect on alternative splicing. Hence, the net effect of IGF2BP3 may be multipronged—with a strong impact on steady-state mRNA levels and an additional impact on splicing—in leukemia stem and progenitor cells.

## Discussion

Here, we have shown the central importance of the RBP IGF2BP3 in MLL-AF4-driven leukemia. MLL-AF4-driven leukemogenesis is characterized by massive transcriptional dysregulation [8]. We confirm here that *Igf2bp3* is a direct transcriptional target MLL-AF4. Interestingly, we determined that IGF2BP3 itself seems to positively regulate MLL-AF4 transcriptional targets. Together, these data suggest that IGF2BP3 and MLL-AF4 form a novel posttranscriptional feed-forward loop, enhancing leukemogenic gene expression. It is not clear if IGF2BP3 may play a role in other leukemia subtypes given its relatively restricted pattern of expression in MLL-translocated leukemia. However, IGF2BP3 overexpression is noted in a wide range of cancer types—with different oncogenic transcriptional programs—and further work is needed to define whether this paradigm may be operant in other hematologic and nonhematologic cancer.

Our prior work showed that IGF2BP3 is required for B-ALL cell survival and overexpression in BM of mice leads to a pathologic expansion of HSPCs [22]. Here, we found that deletion of *Igf2bp3* in MLL-Af4 leukemia caused a striking delay in leukemia development and significantly increased the survival of MLL-Af4 mice. Furthermore, *Igf2bp3* deficiency greatly attenuated the aggressiveness of leukemic disease. Given that MLL-Af4 drives an AML in mice [32], our current work suggests that IGF2BP3 is a powerful modulator of the leukemic phenotype in the myeloid lineage, in addition to the previously observed effects in human B-ALL cells. The lineage of the leukemia induced by *MLL-Af4* in mice may be a limitation of the study, as we cannot conclude an in vivo function for *Igf2bp3* in murine B-ALL. However, Lin et al. showed that there were important pathogenetic similarities between the MLL-Af4-induced pro-B-ALL and AML in mice [32]. In this light, *MLL-AF4* leukemia in humans often shows lineage infidelity and plasticity, which has led to difficulties with targeted therapy [2, 60, 61]. We propose that IGF2BP3 may prove to be a valuable therapeutic target in *MLL-AF4* leukemia, given its function in the pathogenesis of this unique molecular subtype of acute leukemia.

In this study, *Igf2bp3* regulated the numbers and function of LICs. Importantly, the effect of *Igf2bp3* deletion was restricted to LICs and did not significantly impact normal HSC function. Deletion of *Igf2bp3* led to an MLL-Af4 LIC disadvantage in vivo and in vitro. LICs have been defined as cells that can self-renew and have the capability to produce downstream bulk leukemia cells, and their persistence is thought to contribute to relapse after treatment in several different leukemia subtypes [62]. However, the details of human LICs in MLL-AF4 leukemia are less well known [28, 63]. The role of IGF2BP3 in such cells and in relapse of leukemia is of great interest and a future direction for our work.

Previously, we discovered IGF2BP3 interacts primarily with the 3′UTR of target transcripts via iCLIP-seq [22]. Unexpectedly in this study, we determined IGF2BP3 targets transcripts within intronic regions and splice sites in addition to the 3′UTR. These findings may result from utilizing the improved eCLIP technique and the implementation of the technique on primary cells instead of cell lines. Of note, a recent study showed IGF2BP3 may regulate alternative splicing of PKM in lung cancer [64]. We also found IGF2BP3-dependent dynamic splicing events, including retained introns, alternative 3′ss, and skipped exons. Intron retention has been reported to be a mechanism of transcriptome diversification in cancer and, specifically, leukemia [65, 66]. Moreover, studies have highlighted the importance of splicing to mRNA export, and that splicing factor mutations, such as those in U2AF1, result in translational misregulation in myeloid malignancy [67, 68]. Our unexpected, novel discovery, together with our prior work, shows that IGF2BP3 likely regulates specific mRNA operons and functions at multiple posttranscriptional levels, as has been described for other RBPs [69].

As an RBP, IGF2BP3 function is intimately connected to the underlying transcriptional program—IGF2BP3 can only act on specifically induced transcripts in the cell type where it is expressed. Hence, the unique gene sets that are bound and regulated by IGF2BP3 in Lin− and CD11b+ cells are not entirely unexpected, given that transcription changes as LICs differentiate into bulk leukemic cells. This is similar to miRNAs, which posttranscriptionally regulate distinct gene expression programs in distinct cell types [70]. The significant enrichment of IGF2BP3-bound mRNAs in differentially regulated and differentially spliced transcripts confirms a direct regulatory effect. However, further work is required to confirm functional relationships between the specific transcripts that are regulated and the phenotypic effects driven by IGF2BP3.

IGF2BP3 differentially regulated transcripts included MLL-AF4 target genes *Hoxa9, Hoxa10, Hoxa7*, and *Cd69* [32]*. HOXA9*, *HOXA10*, and *HOXA7* are induced by MLL-AF4 and *HOXA9* is required for MLL-rearranged leukemia survival [71]. We determined significant downregulation of both alternatively spliced and full-length isoforms for *Hoxa9*, *Hoxa7*, and *Cd69*. The relationship between leukemogenesis and splicing regulation is complex—while *Hoxa9T*, the homeodomain-less splice variant, is not sufficient for transformation alone, it is required with full-length *Hoxa9* for leukemogenic transformation [59, 72]. Thus, *Igf2bp3* may act through alteration of splicing regulation and upregulation of MLL-Af4 target leukemogenic genes to promote leukemogenesis and impact MLL-Af4 LIC function. Importantly, *Igf2bp3* is not required for steady-state hematopoiesis, in contrast to HOXA9, and may represent a more attractive therapeutic target.

In addition, we found that IGF2BP3 targets and modulates the expression of many transcripts within the Ras signaling pathway and its downstream effector pathways. RAS proteins control numerous cellular processes such as proliferation and survival and are amongst the most commonly mutated genes in cancer [73]. Interestingly, while *MLL-AF4* leukemia has a paucity of additional mutations, the mutations that are present are found in the RAS signaling pathway [74]. In addition, MEK inhibitors have shown selective activity against *MLL*-rearranged leukemia cell lines and primary samples [75]. Hence, IGF2BP3 regulates multiple pathways known to be important in *MLL-AF4* leukemia.

Here, we determined *Igf2bp3* is required for the efficient initiation of MLL-Af4-driven leukemia and function of LICs. Mechanistically, IGF2BP3 binds to hundreds of transcripts and modulates their expression through posttranscriptional mechanisms including regulation of steady-state mRNA levels and pre-mRNA splicing. We describe a novel positional bias for IGF2BP3 binding in leukemic cells isolated from an in vivo model, a notable advance in the field. In summary, IGF2BP3 is an amplifier of leukemogenesis by targeting and regulating the leukemic transcriptome initiated by MLL-AF4, thereby controlling multiple critical downstream effector pathways required for disease initiation and severity. Our findings highlight IGF2BP3 as a necessary regulator of *MLL*-*AF4* leukemia and a potential therapeutic target for this disease.

## Supplementary information

Supplemental Data, Tables, and Methods

Table S3

Table S4

## Data Availability

Data have been deposited onto the NCBI Gene Expression Omnibus repository (GSE156115).
